# Comparative Analysis of a Human Neutralizing mAb Specific for SARS-CoV-2 Spike-RBD with Cilgavimab and Tixagevimab for the Efficacy on the Omicron Variant in Neutralizing and Detection Assays

**DOI:** 10.3390/ijms241210053

**Published:** 2023-06-13

**Authors:** Margherita Passariello, Speranza Esposito, Lorenzo Manna, Rosa Rapuano Lembo, Immacolata Zollo, Emanuele Sasso, Felice Amato, Claudia De Lorenzo

**Affiliations:** 1 Department of Molecular Medicine and Medical Biotechnology, University of Naples “Federico II”, Via Pansini 5, 80131 Naples, NA, Italy; margherita.passariello@unina.it (M.P.); lorenzo.manna@unina.it (L.M.); emanuele.sasso@unina.it (E.S.); 2 Ceinge—Biotecnologie Avanzate s.c. a.r.l., Via Gaetano Salvatore 486, 80145 Naples, NA, Italy; espositosp@ceinge.unina.it (S.E.); rosa.rapuano@unimi.it (R.R.L.); immacolata.zollo@unina.it (I.Z.); 3 European School of Molecular Medicine, University of Milan, 20122 Milan, MI, Italy

**Keywords:** COVID-19, SARS-CoV-2 variants, monoclonal antibodies, immunotherapy, diagnostic approaches, viral neutralization, pseudovirus, combinatorial treatments

## Abstract

The recent pandemic years have prompted the scientific community to increasingly search for and adopt new and more efficient therapeutic and diagnostic approaches to deal with a new infection. In addition to the development of vaccines, which has played a leading role in fighting the pandemic, the development of monoclonal antibodies has also represented a valid approach in the prevention and treatment of many cases of CoronaVirus Disease 2019 (COVID-19). Recently, we reported the development of a human antibody, named D3, showing neutralizing activity against different SARS-CoV-2 variants, wild-type, UK, Delta and Gamma variants. Here, we have further characterized with different methods D3’s ability to bind the Omicron-derived recombinant RBD by comparing it with the antibodies Cilgavimab and Tixagevimab, recently approved for prophylactic use of COVID-19. We demonstrate here that D3 binds to a distinct epitope from that recognized by Cilgavimab and shows a different binding kinetic behavior. Furthermore, we report that the ability of D3 to bind the recombinant Omicron RBD domain in vitro results in a good ability to also neutralize Omicron-pseudotyped virus infection in ACE2-expressing cell cultures. We point out here that D3 mAb maintains a good ability to recognize both the wild-type and Omicron Spike proteins, either when used as recombinant purified proteins or when expressed on pseudoviral particles despite the different variants, making it particularly useful both from a therapeutic and diagnostic point of view. On the basis of these results, we propose to exploit this mAb for combinatorial treatments with other neutralizing mAbs to increase their therapeutic efficacy and for diagnostic use to measure the viral load in biological samples in the current and future pandemic waves of coronaviruses.

## 1. Introduction

Following the emergence of Severe Acute Respiratory Syndrome Coronavirus 2 (SARS-CoV-2), in Wuhan, China, in early 2020, responsible for a disease known as COVID-19 that rapidly spread worldwide, the scientific community has been alerted to develop new therapeutic and diagnostic tools [1]. Although various therapeutic strategies were tested, none was considered to be the gold standard for the treatment of the SARS-CoV-2 infection (with the exception of vaccines approved for preventing the infection). Furthermore, the appearance of antigenically distinct variants showing mutations in the viral Spike glycoprotein, responsible for recognition of receptor human angiotensin-converting enzyme 2 (ACE-2) on the cell surface, has further worsened both the viral detection and the development of therapeutics [2].

Considering the high specificity and selectivity of monoclonal antibodies (mAbs), which target Spike and neutralize the virus, their use in the early phase of infection has shown to be an important therapeutic option in COVID-19 patients [3,4], especially for those with immunodeficiency.

In particular, antibodies directed to the receptor binding domain (RBD) of the Spike protein can interfere in the binding to the ACE-2 receptor on the target host cell and the following infection [5].

Most of currently approved monoclonal antibodies for SARS-CoV-2 are intended as therapeutic for infected immunocompromised patients with severe COVID-19 symptoms or in people with certain medical conditions that cannot count on vaccination. The approved mAbs for therapy of patients infected by SARS-CoV-2 were the following: ‘REGN-COV2’ by Regeneron/Roche, a combination of two non-competing neutralizing IgG1 mAbs with unmodified Fc regions which bind to two distinct and non-overlapping epitopes on RBD, called Casirivimab and Imdevimab [6]. This combination was reported to reduce viral load and was authorized by Food and Drug Administration (FDA) in the US in November 2020 [7].

In February 2021, the FDA awarded the Emergency Use Authorization (EUA) to another combination of therapeutic antibodies, Bamlanivimab plus Etesevimab, developed by Eli Lilly [8,9,10]. In May 2021, the FDA issued the EUA for Sotrovimab, developed by GlaxoSmithKline (GSK), for treatment of mild-to-moderate COVID-19 patients with positive results of SARS-CoV-2 viral testing and at high risk of hospitalization and progression to severe COVID-19 [11]. Then, in December 2021, the European Medicines Agency (EMA) recommended granting marketing authorization to Sotrovimab in the EU [12].

On the other hand, monoclonal antibodies for prophylactic use in high-risk individuals has moved slowly to the clinic. Beyond inborn immune deficiencies (primary), the majority of high-risk patients become susceptible to severe SARS-CoV-2 infection due to acquired immunocompromising diseases (e.g., HIV) or due to comorbidities such as obesity, multiple sclerosis, rheumatoid arthritis, cardiac diseases and cancer where vaccines do not always trigger an effective protective immune response. Only one cocktail of therapeutic antibodies, called ‘Evusheld’, was developed for prophylactic use by AstraZeneca and consisted of a combination of two Fc-modified human mAbs, Tixagevimab and Cilgavimab, formerly called AZD744. Interestingly, this cocktail was approved for prophylaxis of COVID-19 in adults, pediatrics and older patients, and was authorized only for individuals not previously exposed and not infected by the SARS-CoV-2 virus [13].

Unfortunately, several SARS-CoV-2 variants became dominant in many countries, including five variants of concern (VOCs): Alpha, Beta, Gamma, Delta and Omicron. In August 2022, Omicron was classified into five main lineages, BA.1, BA.2, BA.3, BA.4 and BA.5 and some sub lineages (BA.1.1, BA.2.12.1, BA.2.11, BA.2.75, BA.4.6), which, compared to the previous VOCs, harbor more than 30 mutations in the Spike protein with 15 mutations located in the receptor-binding domain (RBD) [14]. As a result of this, not all these antibodies remained effective, because some of them were found to lose the binding ability to the new variants [15,16].

While the interest for COVID-19 pandemic in healthy individuals is waning, there is still a clear and urgent need to accelerate the discovery and clinical translation of subvariants cross-reactive mAbs for prophylactic passive immunization in high-risk individuals. Thus, the evaluation of additional potent and broadly active monoclonal antibodies that can be used to prevent infection with SARS-CoV-2 is still of interest.

To this aim, it could be useful to identify novel specific mAbs binding to unmutated epitopes of novel VOCs Spike-RBD or to combine different mAbs in order to potentiate their antiviral efficacy against new emerging variants. Here, we further characterized D3, a novel human mAb previously generated in our laboratory and characterized for its activity against the wild-type virus (Wuhan, China), UK, Delta and Gamma variants [17,18]. The aim was to investigate its ability to recognize also the other emerged Omicron subvariants to potentiate the therapeutic efficacy in combination with the approved mAbs or to exploit it for potential diagnostic applications.

Hence, we investigated on the ability of novel D3 mAb to recognize Omicron-derived RBD subvariants, either used as purified protein or expressed on pseudoviral particles in vitro, in comparison with the latter antibodies entered in clinical use for treatment of patients infected by SARS-CoV-2, such as Cilgavimab or Tixagevimab. Indeed, we previously compared its biological properties with Sotrovimab, Casirivimab and Imdevimab [18].

Since anti-Spike mAbs, used individually, cannot efficiently block the virus cell entry especially in the case of the Omicron variant, we also investigated the possibility to combine D3 with Cilgavimab by analyzing whether they recognize distinct epitopes on the protein.

Furthermore, using pseudoviral particles in vitro, we also considered harnessing D3 for diagnostic applications in order to detect Spike-protein levels in biological samples.

## 2. Results

### 2.1. Binding to Spike-Omicron Variants of Novel D3 mAb Compared to Those of the Antibodies in Clinical Use for Prophylaxis and Therapy of COVID-19

As previously reported [19,20], different monoclonal antibodies have been approved in the last two years for therapy of SARS-CoV-2, but some of them, which bind to the receptor-binding motif (RBM) of the Spike protein with an affinity in the picomolar range, partially lost their anti-viral activity against the Omicron variant [6,7,21,22,23].

**Figure 1:**
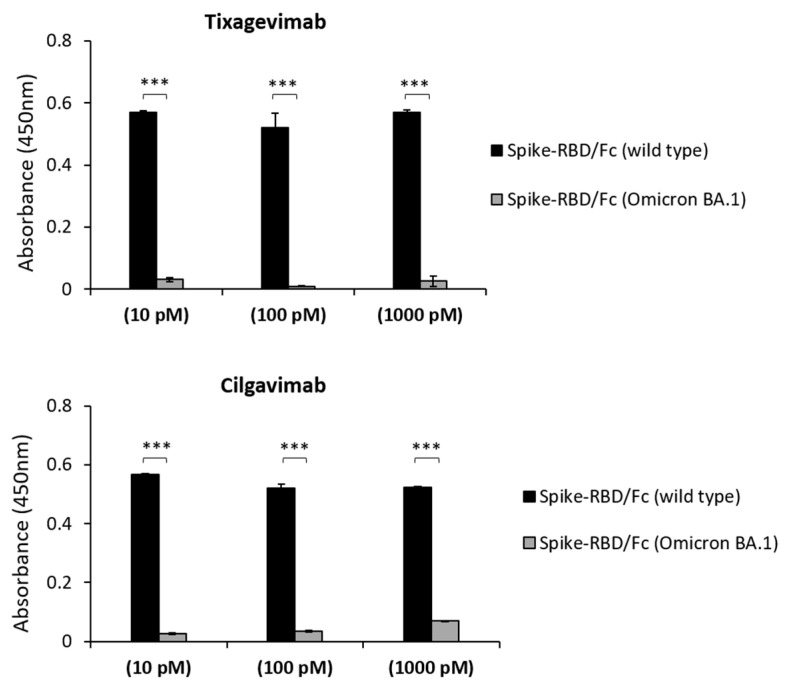
Parallel ELISA assays to analyze the binding of the indicated mAbs on wild-type or Omicron BA.1 variant Spike-RBD. Wild-type (black bars) or Omicron BA.1 (grey bars) rRBD proteins were immobilized, in parallel, on 96-well plates and incubated with Tixagevimab or Cilgavimab at concentrations of 10–1000 pM. The signals were detected by using the human anti-Fab-HRP conjugated antibody and the absorbance values were reported as the mean of at least three determinations. Error bars depicted means ± SD. *p* value: *** *p* ≤ 0.001.

We first analyzed the binding of Tixagevimab and Cilgavimab (10–1000 pM) to the recombinant Spike-wild-type or Omicron-BA.1-derived RBD protein immobilized on nunc-96 well plates. We found that the high affinity binding of these mAbs to the Omicron variant is not retained, as no significant signals were observed at pM and low nM concentrations (Figure 1). Thus, we tested them at higher concentrations in comparison with D3 mAb, which was reported to retain the ability to bind the Spike-Omicron variant in the nanomolar range [17,18].

**Figure 2:**
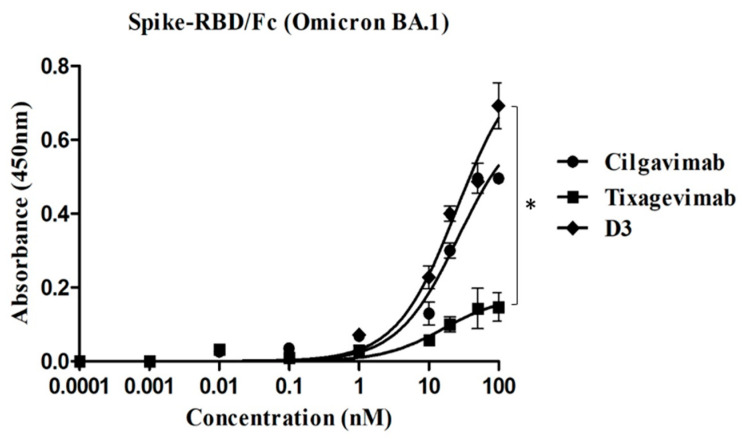
Binding curves on recombinant Spike-RBD Omicron BA.1 protein to compare the affinity of D3 to those of Cilgavimab and Tixagevimab. ELISA assays were performed by evaluating the binding of D3, Tixagevimab or Cilgavimab incubated at increasing concentrations (0.1–100 nM) on immobilized human recombinant Omicron protein. The binding values were reported as the mean of at least three determinations. Error bars depicted means ± SD. *p* value: * *p* < 0.05.

To shed light on this aspect, we tested the three mAbs at increasing concentrations, and we obtained their binding curves on recombinant Spike-RBD Omicron BA.1 protein. D3 mAb confirmed its previous affinity to be in a nanomolar range (25 nM vs. 7 nM for wild-type Spike) [18], comparable to that of Cilgavimab (kd ≈ 25 nM), whereas Tixagevimab showed only a poor binding (Figure 2).

**Figure 3:**
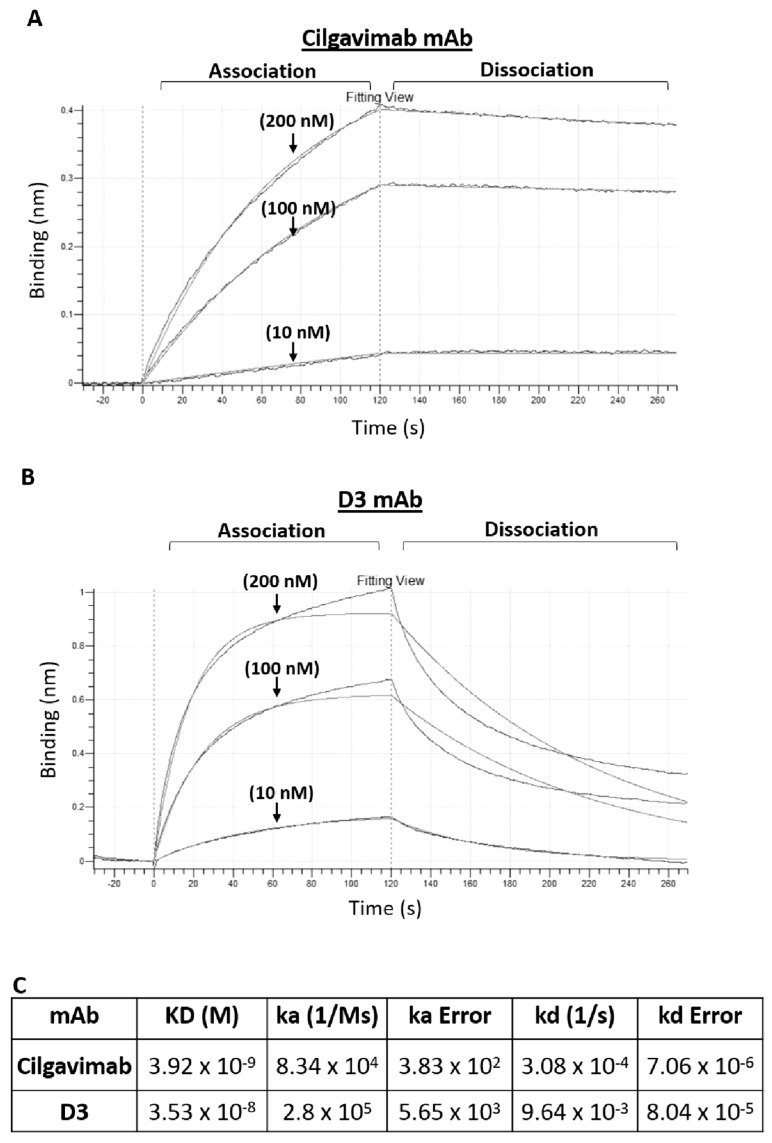
Binding of recombinant Spike-RBD Omicron BA.1 on immobilized D3 or Cilgavimab by BLI. The sensorgrams reported in (**A**) (Cilgavimab) or (**B**) (D3) were obtained by BLI analysis carried out by using Cilgavimab or D3 as ligand and Omicron BA.1 as analyte, tested at increasing concentrations of 10, 100 and 200 nM. The sensorgrams show the rate of association and dissociation of the analyte. (**C**) reports the kd and kinetics of the binding of analyte to the immobilized ligand on a biosensor (protein-A) processed according to the indicated formula A + B $\overset{\mathrm{ka}}{\underset{\mathrm{kd}}{⇄}}$ AB where A represents the analyte and B represents the immobilized ligand, and ka and kd are the association and dissociation rate constants [24]. The data fit uses the 2:1 heterogeneous ligand binding model (by Octet analysis studio 13.0 Software—Biomolecular Binding Kinetics Assays on the Octet^®^ BLI Platform. Apiyo D., Sartorius, Fremont, CA, USA).

To further compare the binding properties and kinetics of Cilgavimab and D3 with Omicron-RBD, we used a real time methodology based on Biolayer Interferometry (BLI). The experimental system consisted of bivalent Cilgavimab or D3 mAb immobilized on the sensor surface with the analyte Spike-Omicron BA.1 (a monovalent analyte) added at increasing concentrations. The kinetic constants for association (ka) and dissociation (kd) were determined. Figure 3 shows the binding curves used to determine ka and kd (see Figure 3C) for the complexes of Cilgavimab or D3 with Omicron-RBD (BA.1 variant). These analyses highlight the different kinetic behaviors for the two antibodies. Cilgavimab binds to Omicron-RBD with a relatively low value of ka (8 × 10^4^ M^−1^ s^−1^), about four-fold lower than the value determined for D3⁄Omicron-RBD complex (3 × 10^5^ M^−1^ s^−1^). With regard to the dissociation step, the Cilgavimab⁄RBD complex was found to be more stable, with a kd value of (3 × 10^−4^ s^−1^), about 30-fold lower than the kd value of D3⁄RBD complex (9.6 × 10^−3^ s^−1^). The calculated equilibrium dissociation constants (kD) for Cilgavimab/RBD and D3⁄RBD complex were 3.9 and 35 nM, respectively (see Figure 3C). The different kD values obtained by BLI, with respect to those calculated by ELISA (25 nM for both the mAbs), are likely due to the analysis of the analyte monovalent binding to the single mAb site (0.5:1) differently from the bivalent equilibrium binding of mAbs to the immobilized target measured by ELISA, which is also influenced by avidity.

**Figure 4:**
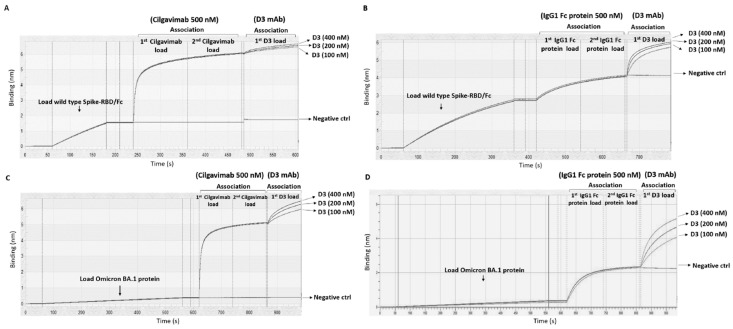
Competitive Binding of D3 and Cilgavimab on wild-type or Omicron BA.1 derived Spike-RBD protein by BLI analyses. (**A**–**C**) The binding curves reported in the sensorgrams A and C show the association of Cilgavimab, injected two times at saturating concentration (500 nM) and followed by incubation with D3 used at increasing concentrations (100–400 nM), to parental (**A**) or Omicron BA.1-derived Spike-RBD protein (**C**), used as the immobilized ligand. As a control, D3 was tested in parallel on the immobilized protein in the absence of Cilgavimab reported as negative control. (**B**,**D**) In these control experiments, IgG Fc recombinant protein was injected two times at the concentration of 500 nM and followed by D3 used at increasing concentrations (100–400 nM).

The competition—or lack thereof—between Cilgavimab and D3 for binding to Spike-RBD was further tested by Biolayer Interferometry. To this aim, D3 was added over Spike-Fc immobilized on protein A-sensor, before and after saturation with two incubations of 500 nM Cilgavimab (Figure 4). After the ligand on the surface was saturated by repeated additions of Cilgavimab until no significant additional response was observed (Figure 4), increasing amounts of D3 were tested. Binding curves of D3 were observed, indicating that the antibody could bind to Spike on the sensor surface even after saturation with Cilgavimab. To further confirm this result, we repeated the same experiment by using immobilized Omicron BA.1-derived Spike-RBD. After the saturation with two following incubations of 500 nM Cilgavimab, D3 was added at increasing concentrations showing the ability to bind to the Omicron BA.1 variant RBD even when saturated with Cilgavimab. As a control, the experiments were repeated in the absence of Cilgavimab by using the only Fc region (two following incubations at 500 nM) to saturate the pro A or pro G biosensor (see Methods) before the addition of D3. The binding of D3 in these conditions was higher than that observed in the presence of Cilgavimab. These data indicate that the two mAbs bind to distinct or only partially overlapping epitopes of Spike-RBD.

Furthermore, when Cilgavimab was tested by ELISA for its ability to recognize the immobilized peptide sequence previously recognized by D3 [18], no binding was observed at concentrations up to 300 nM), thus confirming that it recognizes an epitope different from that of D3.

**Figure 5:**
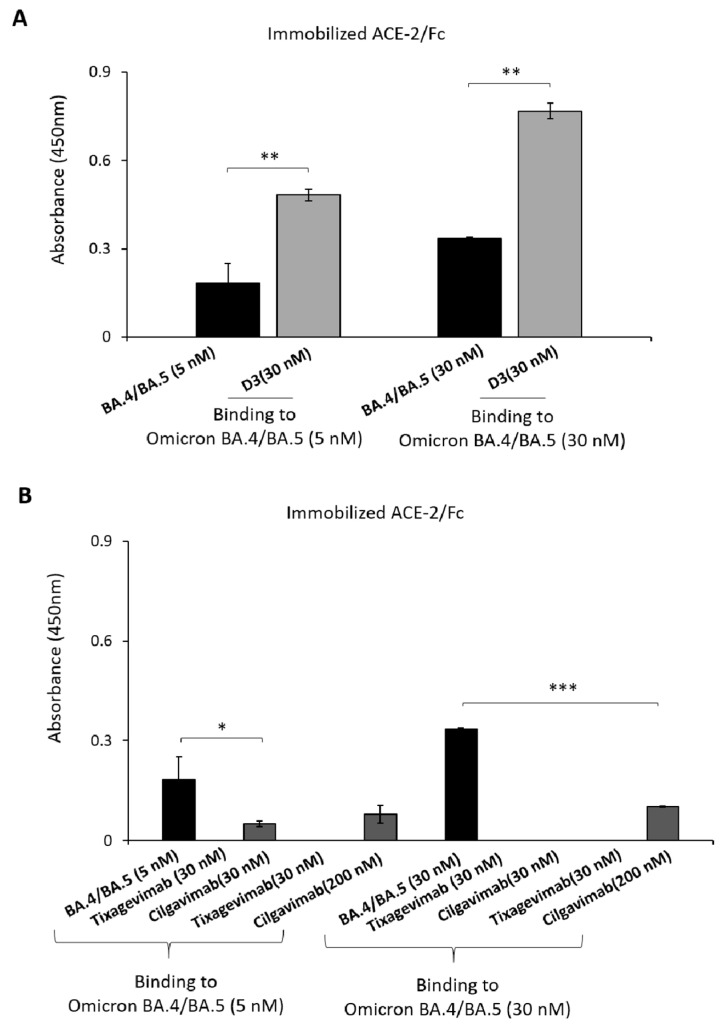
Binding of D3, Cilgavimab or Tixagevimab to Spike-RBD derived from Omicron BA.4/5 variant by ELISA assays. ACE-2/Fc protein was immobilized on 96 well plate (5 ug/mL) and preincubated with the Omicron BA.4/5-His variant, used at 5 or 30 nM, for 90 min at 25 °C. After extensive washes, D3 mAb (**A**), as indicated by the grey bars, was added at the concentration of 30 nM whereas Tixagevimab or Cilgavimab (**B**) were added at the concentrations of 30 and 200 nM (grey bars). The signals were detected by using the human anti-Fab-HRP conjugated antibody and the absorbance values were reported as the mean of at least three determinations. Error bars depicted means ± SD. *p* values: * *p* < 0.05, ** *p* < 0.01, *** *p* ≤ 0.001.

Finally, we analyzed the binding of D3, Tixagevimab and Cilgavimab to the latest emergent Omicron BA.4/5 variant [14] by ELISA assays. Briefly, ACE-2 receptor was immobilized on the plates, then incubated with Omicron BA.4/5 RBD-His at two different concentrations (5 and 30 nM) and detected by using either D3 or Cilgavimab followed by a HRP-conjugated anti-Fab antibody, or by an HRP-conjugated anti-His mAb, used as a positive control (Figure 5).

We found that D3 recognizes the recombinant protein in a dose-dependent fashion when used at a concentration of 30 nM, whereas Tixagevimab and Cilgavimab did not significantly bind to the protein when used at this or even higher concentrations up to 200 nM.

### 2.2. Evaluation of the Anti-Spike mAbs for Diagnostic Applications

Once the binding affinity of D3 for the Omicron-RBD variants in comparison with the antibodies in clinical use was confirmed, we also decided to investigate the possibility of using this novel mAb for diagnostic applications.

**Figure 6:**
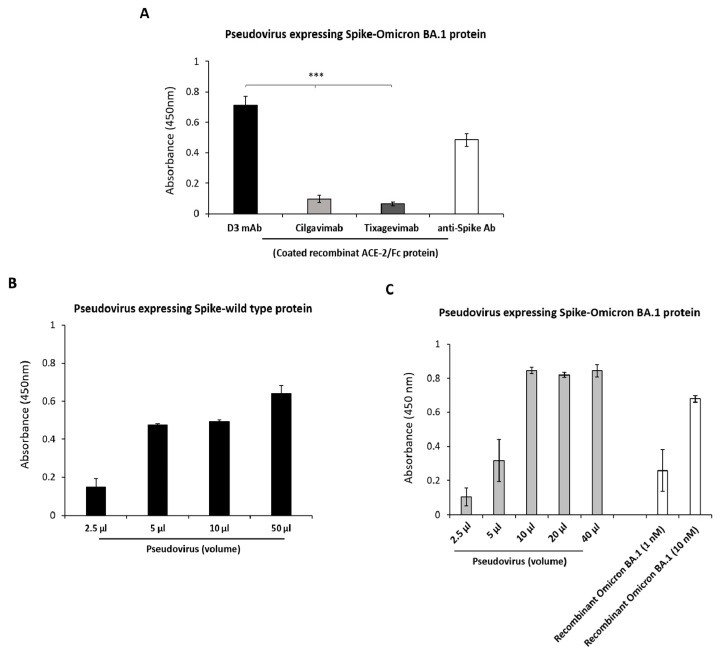
Detection of Spike-RBD protein expressed on pseudoviruses by D3 compared to the mAbs approved for treatment of COVID-19. (**A**) ACE-2/Fc recombinant protein was immobilized on 96-well plate and pre-incubated with the supernatant containing pseudoviral particles (4000 particles/μL) expressing Spike-Omicron BA.1 protein. D3 or the other indicated mAbs were added at the concentration of 30 nM and the signals were detected by using an anti-Fab HRP-conjugated antibody. (**B**,**C**) D3 was used to detect pseudovirus expressing wild-type protein (black bars) or Omicron BA.1 protein (grey bars) incubated at increasing concentrations on immobilized ACE-2/Fc protein. Recombinant purified Spike-Omicron BA.1 protein (white bars) was used at two different concentrations as a positive control. Binding values were reported as the mean of at least three determinations. Error bars depicted means ± SD. *p* value: *** *p* ≤ 0.001.

To this aim, D3 was analyzed, in parallel with Cilgavimab or Tixagevimab, as a tool to detect the level of Spike protein and consequent viral load in biological samples by setting up an assay based on the use of pseudoviruses expressing Spike-Omicron BA.1 protein. Briefly, ACE-2 receptor, used as a capture molecule, was immobilized on a 96-well plate; the supernatant recovered by HEK293T-Pseudovirus packaging cells, as described in Materials and Methods, was added. D3, Cilgavimab or Tixagevimab mAb, at the concentration of 30 nM, was then added to detect the binding to the protein expressed on pseudoviral particles. The anti-Spike commercial Ab was also included as a control. The results, reported in Figure 6A, show that D3, in line with its ability to bind to the Omicron-RBD [18], also recognized the Omicron Spike protein-expressed on pseudoviruses with satisfactory efficiency, whereas Cilgavimab and Tixagevimab did not show significant binding. On the basis of these encouraging results, the assays were repeated by testing D3 on pseudoviral particles, expressing either wild-type or Omicron-derived RBD, used at increasing concentrations. The results, reported in Figure 6, showed that D3 efficiently recognized pseudoviral particles expressing either D614G- or Omicron BA.1 in a dose-dependent manner, with the lowest signal obtained by using 10,000 pseudoviral particles. Hence, D3 is able to recognize Spike on viral-like particles and could be used to measure viral load in biological samples.

### 2.3. Neutralization Assays Using SARS-CoV-2 Spike-Pseudotyped Lentivirus

The neutralizing activity of D3 against the wild-type virus and the UK, Delta and Gamma variants of SARS-CoV-2 was previously reported [17,18]. Here, we verified whether its neutralizing activity was retained also against the latter OmicronBA.1 variant.

Firstly, we analyzed the ability of D3 to interfere in the interaction of ACE-2 and Omicron-RBD by BLI analyses. Briefly, ACE-2 Fc was loaded on a protein A sensor, then, Omicron BA.1-RBD was added as analyte at a concentration of 50 nM before or after an incubation of 1 h with a molar excess of D3, Cilgavimab and an unrelated mAb, used as controls.

**Figure 7:**
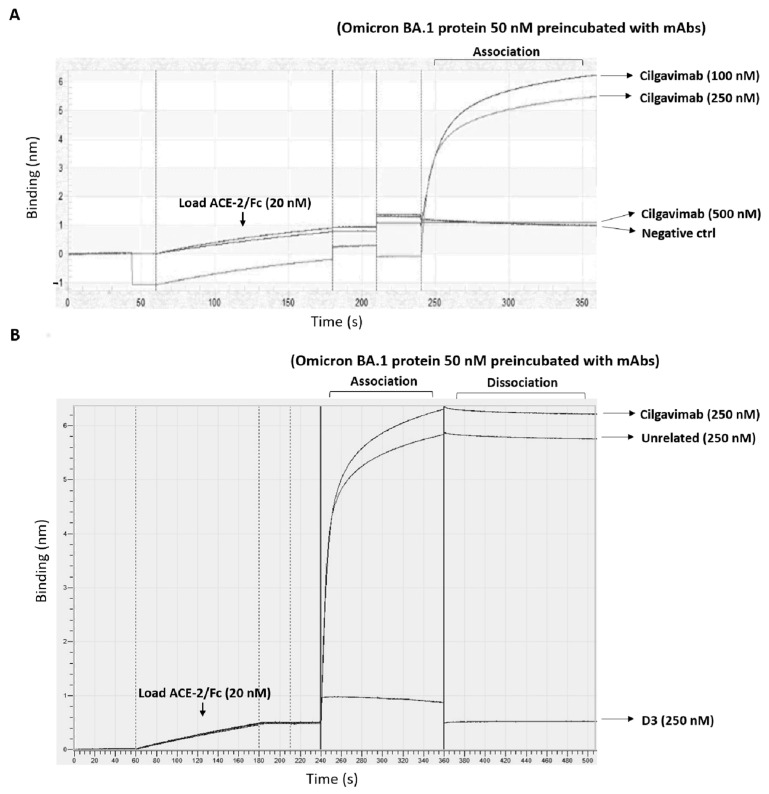
Binding of Spike-RBD Omicron BA.1 to ACE-2 receptor, in the absence or in the presence of D3 and Cilgavimab mAbs, by BLI. (**A**) The sensorgrams show the association of the Omicron BA.1 analyte (50 nM) to ACE-2/Fc, used as ligand, before or after pre-incubation of 1 h with increasing concentrations of Cilgavimab. (**B**) The association of Omicron BA.1 analyte, reported in the sensorgrams, was evaluated after a preincubation of 1 h at 25 °C with an unrelated mAb, used as a negative control, Cilgavimab or D3 at the indicated concentrations.

As shown in Figure 7, D3 fully blocked the association of the two partners at a concentration of 250 nM (5:1 molar excess) whereas Cilgavimab was not able to inhibit the association when used at concentrations of 100 and 250 nM, but it was blocking the complex formation at the higher concentration of 500 nM. As expected, the unrelated mAb did not interfere with the association, thus confirming the specificity of the other two mAbs for Spike.

Thus, to evaluate the anti-viral efficacy of mAbs of interest we performed a neutralization assay using a SARS-CoV-2-pseudotyped virus.

**Figure 8:**
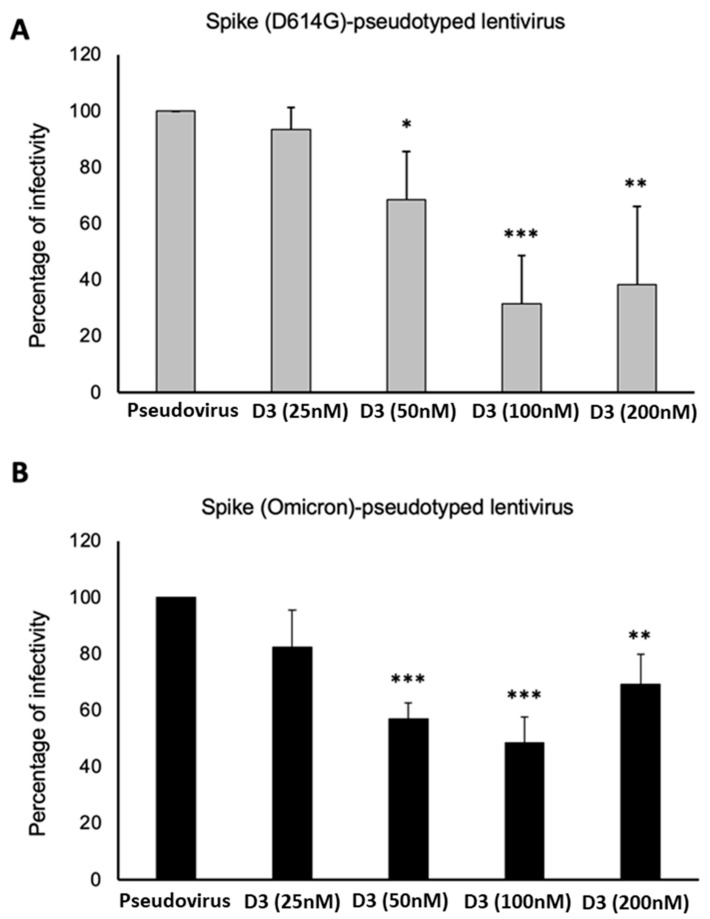
Neutralization activity of D3 on wild-type or Omicron BA.1-derived pseudoviruses in cell cultures. Pseudoviruses expressing D614G- (**A**) or Omicron- (**B**) Spike protein were pre-incubated with D3 mAb, used at increasing concentrations (25–200 nM), for 1 h at 37 °C. Then, HEK293-ACE2 cells were infected with the mixtures for 48 h and the levels of infectivity expressed as percentage of luminescence with respect to untreated infected cells used as a positive control. Error bars depicted are means ± SD and were obtained by three independent experiments. *p*-value: * *p* < 0.05, ** *p* < 0.005, and *** *p* < 0.0005 by unpaired two-tailed Student’s *t*-tests.

In particular, D3 mAb was used at increasing concentrations (25–200 nM) and preincubated with the pseudoviral particles for 1 h at 37 °C. Then, the mixtures were added to the ACE-2-expressing HEK293 cells and incubated for 48 h at 37 °C. After the incubation, the putative neutralizing effect of D3 mAb was evaluated, as reported in Methods, by measuring the luciferase activity expressed by infected cells, expressed as percentage with respect to the untreated infected cells (Figure 8). We found that D3 significantly inhibited the infection of the D614G-variant pseudoviruses in a dose-dependent manner, whereas it showed a slightly lower efficiency on the Omicron-derived pseudoviruses, even though it retained the ability to inhibit the infectivity by 50% at 50 nM.

**Figure 9:**
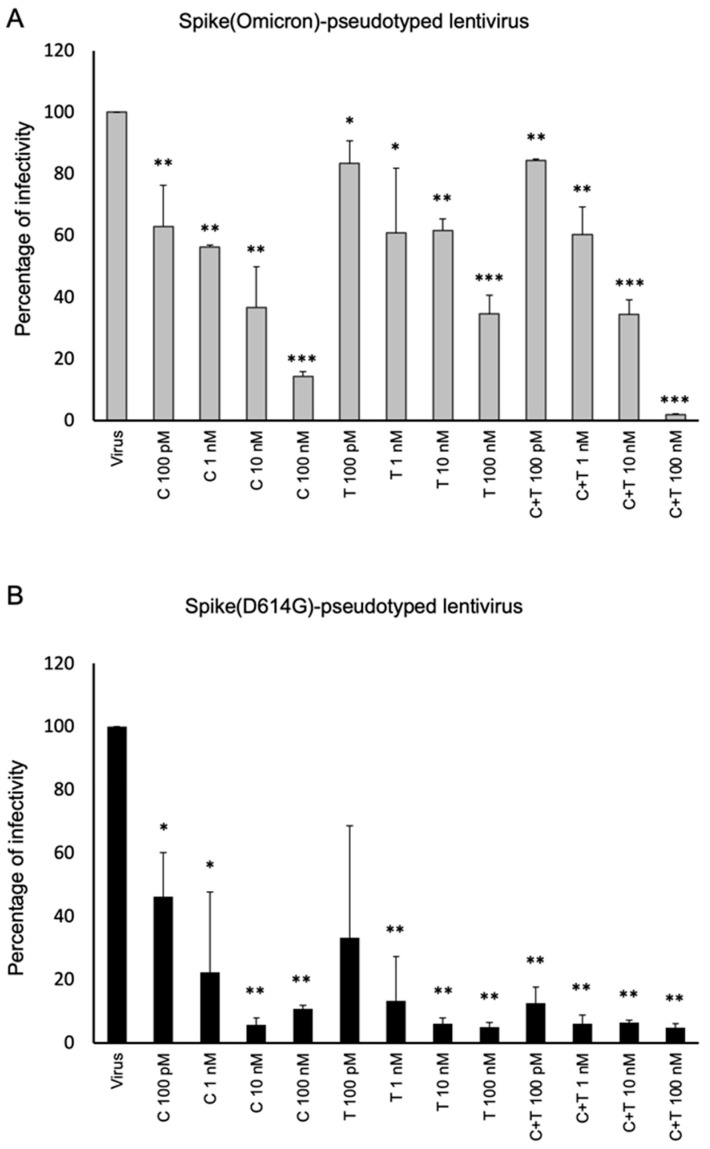
Neutralization activity of Cilgavimab © and/or Tixagevimab (T) on D614G or Omicron-derived pseudoviruses. Pseudoviruses expressing D614G- (**A**) or Omicron- (**B**) Spike protein were pre-incubated with C, T or a combination of C + T, used at increasing concentrations (from 100 pM to 100 nM), for 1 h at 37 °C. The levels of infectivity were expressed as percentage of luminescence with respect to untreated infected cells used as a positive control. Error bars depicted means ± SD. *p*-values: * *p* < 0.05, ** *p* < 0.005, *** *p* ≤ 0.001 by unpaired two-tailed Student’s *t*-tests.

In parallel assays, Cilgavimab was tested in the same conditions alone or in combination with Tixagevimab for comparison. Both mAbs had a higher neutralizing effect versus Spike (D614G)-pseudotyped virus than Omicron BA.1. Between the two mAbs, Cilgavimab was much more effective compared to Tixagevimab (Figure 9).

Their combination increased their neutralizing efficacy against Spike D614G-expressing pseudoviruses, but did not show such beneficial effects against Omicron-Spike-expressing pseudoviruses, with respect to Cilgavimab in monotherapy, except when they were used at the highest concentration (100 nM).

**Figure 10:**
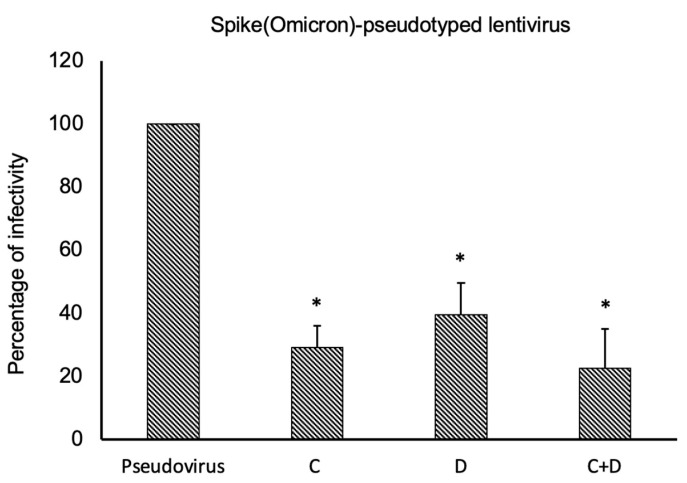
Neutralization activity of Cilgavimab (C), and/or D3 mAbs on Omicron-derived pseudoviruses. Pseudoviruses expressing Omicron-Spike protein were pre-incubated with 50 nM of C, T, and D3 mAbs or combination of them for 1 h at 37 °C. The levels of infectivity were expressed as percentage of luminescence with respect to untreated infected cells used as a positive control. Each error bar depicted is the mean ± SD of three independent experiments. * *p* < 0.005.

Since we demonstrated an effective neutralization after treatment with Cilgavimab, we decided to evaluate whether the efficacy of Cilgavimab against the Omicron BA.1 variant could be potentiated when used in combination with the novel D3 mAb. As shown in Figure 10, D3 and Cilgavimab showed a good efficacy in the inhibition of pseudoviral infectivity; however, their combination did not significantly increase the anti-viral effects with respect to single treatments.

## 3. Discussion

In this study, we show that D3 mAb [17,18] is effective in binding to the RBD domain of SARS-CoV-2 Spike protein and its derived Omicron variants in in vitro assays. Moreover, D3 is also effective in neutralizing the infectivity of Spike-pseudotyped lentivirus expressing the Spike (D614G and Omicron BA.1) protein variant. We have previously identified the epitope recognized by D3 and demonstrated how its localization, upstream of the RBM domain, allows D3 to maintain a good binding capacity towards the different variants of the Spike protein differently from Casirivimab or Imdevimab [18]. Here, we further characterized the ability of D3 to bind the last emerged Omicron subvariants in both in vitro and in-culture based assays compared to Cilgavimab and Tixagevimab, currently in clinical use for COVID-19 prophylaxis and/or treatment [25,26].

We analyzed the ability of D3 to interfere in the interaction of ACE-2 and Omicron BA.1-RBD by BLI analyses, showing that D3 inhibits the recognition of Omicron more efficiently than Cilgavimab. Thus, we further investigated on the epitope recognized by the two mAbs and we found that D3 binds to a distinct epitope of Spike-RBD protein from that recognized by Cilgavimab, as shown by BLI analyses of D3 on immobilized Spike-RBD protein in the presence of saturating concentrations of Cilgavimab.

The binding affinity of D3 to the Omicron BA.1 variant, previously assessed by ELISA [18], was further investigated by BLI analyses, showing that D3 binds with high affinity to this variant and shows a different binding kinetic behavior when compared to Cilgavimab, showing faster association and dissociation rates.

These results led us to also consider D3 to be useful for diagnostic applications in order to detect the Spike-protein levels in biological samples; indeed, we demonstrate here that D3 efficiently and quantitatively detects pseudoviral particles expressing both the D614G Spike protein and its derived Omicron BA.1 variant in ELISA assays. The higher capacity of D3 to bind to pseudoviral particles by ELISA with respect to Cilgavimab could be due to a better accessibility of the epitope recognized by D3 when the protein is expressed on the pseudoviral surface. Considering that the individual virions of SARS-CoV-2 display about 20–30 trimers of the Spike protein on the surface, as reported in the literature, whereas pseudoviral particles likely express only a few copies of Spike, we can assume that detection by D3 of viral particles could be even more sensitive than that observed with pseudoviral particles [27,28].

Furthermore, differently from Cilgavimab, D3 is still able to bind to Omicron BA.4/5 RBD and it likely retains the ability to recognize the new emerging Omicron subvariants, such as BQ (R346T, K444T, N460K) and XBB (N460K, F486P, F490S) [16], as their mutations are located in different regions with respect to that recognized by D3 [18], whereas partially affecting the epitope recognized by Cilgavimab [18].

Additional studies were performed to verify whether D3 also preserved its neutralizing ability in the case of Omicron variant. To this aim, pseudoviral particles expressing the D614G Spike protein or its derived Omicron BA.1 variant were used for neutralization assays. The pseudoviruses were pre-incubated with D3 mAb. Then, ACE2-expressing cells were infected for 48 h before measuring the inhibitory effects. We found that D3 also inhibited the infectivity of pseudoviral particles expressing Omicron-Spike, even though it had a slightly lower efficacy than the neutralization activity observed with the virus expressing the D614G Spike variant. Unfortunately, the combination of D3 with Cilgavimab did not significantly improve the inhibition of Omicron-derived pseudoviruses infectivity with respect to single agent treatments.

Thus, we can conclude that D3 mAb could become a precious tool not only for therapeutic use in combinatorial treatments with other effective mAbs, such as Sotrovimab, but also for diagnostic applications in the current and future pandemic waves of Coronavirus variants, thanks to the ability of D3 to maintain an excellent ability to recognize the Spike protein despite the various variants.

## 4. Materials and Methods

### 4.1. Cell Line Cultures

Human embryonic kidney cells (HEK293T), human embryonic kidney cells expressing human ACE-2 receptor (HEK293–hACE2), were cultured in Dulbecco’s Modified Eagle Media (DMEM) supplemented with 10% (*v*/*v*) fetal bovine serum (FBS), 100 U mL^−1^ of penicillin-streptomycin (Pen-Strep), 1 mM of sodium pyruvate and 2 mM of L-glutamine (all from Euroclone, Milan, Italy). Cell lines were purchased from the American Type Culture Collection (ATCC, Manassas, VA, USA) and were cultured in humidified atmosphere containing 5% CO_2_ at 37 °C.

### 4.2. Antibodies and Human Recombinant Proteins

The following human recombinant proteins were used: human chimeric SARS-CoV-2 (2019-nCoV) Spike-RBD/Fc protein was purchased from Sino Biological (Eschborn, Germany). Human chimeric SARS-CoV-2 BA.1 Omicron (B1.1.529) Spike-RBD/Fc protein was purchased from Sino Biological. Human chimeric SARS-CoV-2 Omicron BA.4/BA.5 Spike-RBD/His protein was purchased from R&D Systems (Minneapolis, MN, USA). Human chimeric ACE-2/Fc protein was purchased from GenScript (Piscataway, NJ, USA). Human Recombinant IgG1 Fc protein was from Abcam (Cambridge, UK).

The following antibodies were used: Tixagevimab and Cilgavimab, two human Fc-modified IgG1 derived from B cells from two individuals who had recovered from SARS-CoV-2 infection and developed by AstraZeneca (Samsung Biologics, Incheon, Republic of Korea). D3 mAb is a fully human IgG4 mAb isolated by phage display, which was expressed and purified as previously described [11].

Human SARS-CoV-2 Spike protein (RBD) polyclonal antibody was purchased from Invitrogen (Rockford, IL, USA). HRP-conjugated anti-human IgG (Fab’)2 goat polyclonal antibody from Abcam (Cambridge, UK) was used for the detection of primary mAbs.

### 4.3. Binding of mAbs to Spike-RBD from Wild-Type SARS-CoV-2 or Its Omicron Variants

To test the binding of D3, Tixagevimab and Cilgavimab to the wild-type Spike-RBD protein or to its derived Omicron variants, ELISA assays were performed as previously described [29,30,31] by incubating the 96-well nunc plates, previously coated with the target proteins (5 µg/mL), with D3, Tixagevimab or Cilgavimab mAbs at increasing concentrations (10–1000 pM) for 2 h at RT. After extensive washes, the HRP-conjugated anti-Fab antibody was added. The binding signal was then detected as previously described [11,12].

### 4.4. Biolayer Interferometry (BLI) Analysis

The BLI analyses were performed by using the Octet^®^ R4 Protein Analysis System (Sartorius, Fremont, CA, USA). Biosensors carrying the protein A (Octet^®^ ProA Biosensors, Sartorius, Fremont, CA, USA) were used to perform the assays.

Prior to the BLI run, the ProA or ProG biosensors tips were hydrated for 15 min in 200 μL of Kinetic Buffer (KB) 10× (0.1% BSA, 0.02% Tween, in PBS 1X). Then, the program steps were set on the BLI software (Octet^®^ BLI Discovery Software 13.0).

The biosensors were loaded with each Spike-RBD/Fc recombinant protein or each anti-Spike monoclonal antibody, used at the concentration of 4 µg/mL, for an interval of time up to 120 s, as the Fc region was found able to saturate the ProA-biosensor in these conditions. After washing, the association step was carried out by incubating the biosensors for 240 s in a solution containing the analytes (RBD-Spike over the immobilized mAb or each mAb (Cilgavimab or D3 over the immobilized Spike-RBD) diluted at increasing concentrations. The dissociation step was performed in KB buffer 10× for 150 s. Finally, the biosensors were regenerated according to manufacturer’s recommendations.

The data were acquired and processed into the Octet^®^ Analysis Studio Software 13.0 to perform the analysis.

### 4.5. Lentiviral Production and Cells Transduction

Lentiviral particles pseudotyped with the SARS-CoV-2 (COVID-19) B.1.1.529 (Omicron) Spike protein variant were packaged in HEK293T cells by using third-generation packaging plasmids provided by Cellecta (Mountain View, CA, USA). All procedures were performed according to the manufacturing protocol.

The cells (4 × 10^6^) were plated in 10-cm plates with 10 mL of media for 24 h prior to transfection and then the Packaging Plasmid Mix (0.5 μg/μL) and plasmids carrying a reporter expressing luciferase and yellow fluorescent protein were co-transfected in cells by using lipofectamine 2000 transfection reagent (ThermoFisher Scientific, Waltham, MA, USA). Medium containing complexes was replaced after 12 h of transfection with 10 mL of fresh DMEM supplemented with 10% FBS, DNase I (1 U/mL), MgCl2 (5 mM) and 20 mM HEPES, pH 7.4. The virus-containing medium was collected after 24, 48 and 72 h post-transfection from each plate and filtered through a Nalgene 0.2 μm PES filter to remove debris and floating packaging cells.

The viral particles pseudotyped with the SARS-CoV-2 (COVID-19) D614G Spike protein were generated by co-transfecting the Lenti-Pac HIV Expression Packaging Kit (GeneCopoeia, Rockville, MD, USA), an optimized mixture of plasmids that expresses the structural, regulatory and replication genes required to produce lentivirus in HEK293T cells. According to the manufacturer’s protocol, 2.5 µg of lentiviral expression plasmid and 5.0 µL (0.5 µg/µL) of Lenti-Pac HIV were mixed and co-transfected with plasmids carrying a reporter expressing luciferase and yellow fluorescent protein by EndoFectin (GeneCopoeia).

The supernatant containing lentiviral particles was used directly to determine the titer by ddPCR and to transduce HEK293–hACE2 expressing cells by incubating them for 48 h. Lentiviral stocks were aliquoted and stored at −80 °C.

### 4.6. Quantization of Viral Particles by Droplet Digital PCR (ddPCR)

To quantify the pseudoviral particles titer, 100 µL of supernatant containing lentiviral particles was mixed to an equal volume of QuickExtract RNA solution (Lucigen, Middleton, WI, USA) and incubated at 95 °C for 5 min. Then, 15 µL of extracted RNA was retrotranscribed to generate cDNA. Then, several dilutions (1:10, 1:100 and 1:1000) of cDNA were assembled in a Digital Droplet PCR reaction with EvaGreen Mastermix (Bio-Rad, Hercules, CA, USA), using a primer pair specific for the luciferase gene (LUC), Forward 5′-AAGAGGCGAACTGTGTGTGAGA-3′ and reverse 5′-ATGTAGCCATCCATCCTTGTCA-3′.

Next, the mix was used to generate droplets (QX200 droplet generator, Bio-Rad) before the PCR reaction. Then, all droplets were read using a QX200 Droplet Reader (Bio-Rad) to determine the number of viral particles using QuantaSoft Software Version 1.7.4.

Droplet Digital PCR thermal cycling conditions used for the assay were indicated in the Table 1 reported below:

### 4.7. In Vitro Neutralization Assays of D614G/Omicron BA.1 SARS-CoV-2 Variant

HEK293–hACE2 cells seeded in 96-multi-well plate at 70% of confluence were infected with the pseudoviral particles at 0.6 MOI for 48 h with a pseudovirus carrying a reporter expressing luciferase.

Pseudoviruses and neutralizing antibodies were preincubated at 37 °C for 1 h before infecting the HEK293–hACE2 cells. After 48 h, cells were lysed with 40 µL of passive lysis buffer (PLB) (Promega, Milan, Italy). To analyze luciferase activity, aliquots of 10 μL of lysate were loaded into a black flat bottom Costar 96-well plate, and 35 µL of Luciferase Assay Reagent II (Dual Luciferase Reporter Assay LAR II, from Promega, Milan, Italy) were dispensed to each well to generate a luminescent signal. The expression of a luciferase reporter was quantified as the luminescence produced above background level by PHERAStar FSX reader, BMG LABTECH (Freiburg, Germany), and analyzed by Mars Data Analysis, FDA 21CFR Software. Non-infected or infected cells with pseudoviruses only were used as negative and positive control, respectively, while the neutralization activity of mAbs was expressed as percentage with respect to positive control.

### 4.8. Detection of Pseudoviral Particles Expressing Wild-Type Spike or Its Omicron Variant

To detect the levels of Spike-RBD protein derived from wild-type or Omicron BA.1 variant, ELISA assays were performed by using supernatants containing pseudoviral particles expressing Spike, derived from cells transduced as described above. ACE-2/Fc recombinant protein was immobilized on nunc 96-well plate and pre-incubated with serial dilutions of supernatants containing pseudoviral particles, and the plates were incubated for 2 h at RT. After washes, D3, Tixagevimab, Cilgavimab or the human anti-Spike-RBD commercial Ab (used as positive control) was added in parallel at a concentration of 30 nM, and the plates were incubated for 2 h at RT. Then, the plates were washed and the signals detected by using an anti-Fab HRP-conjugated antibody, as previously described [32,33,34,35].

### 4.9. Statistical Analyses

All the data are reported as means ± SD. Statistical significance was defined by unpaired two-tailed Student’s *t*-tests and represented as follows: * *p* < 0.05, ** *p* < 0.01, *** *p* ≤ 0.001 [36].

## Figures and Tables

**Table 1:** Droplet digital reaction program.

Step	Temperature	Time	Ramp Rate	Cycles
Initial denaturation	95 °C	10 min	2.5 °C/s	1
Denaturation	95 °C	30 s	40	
Annealing	55 °C	30 s		
Extension	72 °C	15 s		
Signal stabilization	98 °C	10′	1 old (optional) 12 °C	infinite

## Data Availability

All the new data are contained in the present paper.
